# Th17-type immunity and inflammation of aging

**DOI:** 10.18632/aging.203119

**Published:** 2021-05-28

**Authors:** Kristen M. Merino, S. Michal Jazwinski, Namita Rout

**Affiliations:** 1 Division of Microbiology, Tulane National Primate Research Center, Covington, LA 70433, USA; 2 Tulane Center for Aging, Tulane University School of Medicine, New Orleans, LA 70112, USA

**Keywords:** inflammation, Th17-type, IL-17, IL-22, CD161

Chronic sub-clinical inflammation of aging, resulting from lifetime exposures to pathogens in concert with impaired immune responses, poses an obstinate challenge to the health span of the growing elderly population. Older adults are not only at a greater risk for age-related diseases, but also for more severe infections. Of note, pneumonia, influenza, sepsis, and recently severe acute respiratory syndrome coronavirus 2 (SARS-CoV-2) have caused significant mortality among elders. Several factors contribute to the increased morbidity/mortality of older adults, including loss of naïve lymphocytes, exhaustion of adaptive immunity, and a skew toward proinflammatory responses. Additionally, loss of intestinal homeostasis and perturbations in epithelial barrier protective immune functions have recently emerged as key factors underlying chronic inflammation and age-related comorbidities.

Defense of epithelial barriers against invading pathogens and maintenance of mucosal homeostasis mainly relies on the Th17-type immunity, also known as type-17 and Th3 immunity, which is characterized by IL-17/IL-22 cytokine production. IL-17 predominantly triggers the influx of neutrophils and tissue repair, while IL-22 stimulates proliferation of epithelial cells, and regulates epithelial permeability, production of mucus, antimicrobial proteins, and complement to help maintain barrier integrity (Figure 1). Th17-type immunity involves multiple cell types including ILC3/NK cells, γδT cells, MAIT cells, CD4 helper (Th17) and CD8 (Tc17) αβT cells. These cell types are characterized by surface expression of CD161, transcription factors retinoic acid-related orphan receptor (Rorγt) and Aryl hydrocarbon receptor (AhR), and they have been shown to localize at important barrier sites. Th17-type immunity has proved indispensable for fighting off fungal infections and has been shown to protect against *Klebsiella* and *Streptococcus* pneumonia among other mucosal bacterial infections. However, this response has also been implicated in several autoimmune disorders such as ﻿Crohn’s disease, psoriasis and multiple sclerosis [1]. Thus, careful examination of the Th17-type cells within mucosal tissues remains important for determining their specific role in protection or pathogenesis in different disease settings, particularly in the inflammation of aging.

**Figure 1:**
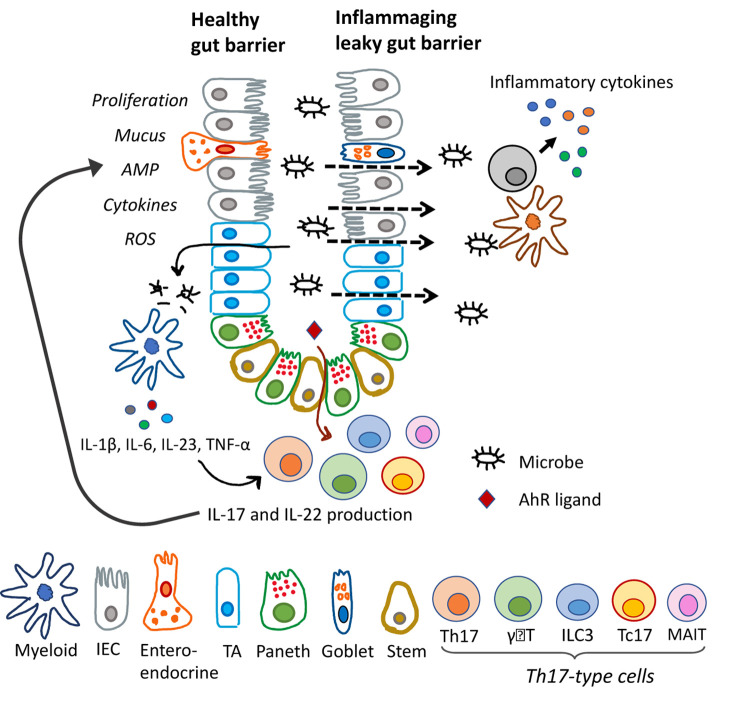
**Role of Th17-type immune responses in intestinal barrier functions and inflammaging.** Schematic representation of gut epithelia with diverse cell types. Microbial signals stimulate IL-17/IL-22 cytokine production, and their effector functions on epithelial cells are shown on the left side. Dysfunctional Th17-type immune response results in leaky gut, microbial translocation and inflammation, as shown on the right side. IEC, intestinal epithelial cell; TA, transit amplifying.

Our laboratory has shown that systemic inflammation in older macaques was associated with reduced Th17-type cytokine functions of CD161+ immune cells [2]. This correlated with circulating biomarkers of leaky gut and microbial translocation, suggesting a link between intestinal barrier dysfunction and inflammaging [2]. Notably, HIV infection is a textbook example of how impaired Th17-type immunity contributes to the massive damage in gut barrier integrity leading to chronic inflammation and HIV-related pneumonia and lung injury. In the macaque model of ART-treated HIV infection, we have shown that following viral suppression and resolution of acute inflammation, loss of IL-22 and IL17 responses in gut mucosal γδT cells led to barrier damage and systemic inflammation [3]. Other studies found that in baboons, mice, and human dendritic cells, the Th17-type response is an important correlate of protection against *Bordetella pertussis*. Mice lacking the IL-17 receptor lost the ability to clear the infection, while combined Th1/Th17 responses triggered long-term *B. pertussis* immunity [4].

While much research is aimed at understanding the protection provided by Th17 cells, other studies have demonstrated disease exacerbation. RSV and SARS-CoV-2 infection studies have linked the overstimulation of Th17-type immunity with severe lung pathologies [5], underscoring the need to find optimal equilibrium between Th17-type cells and T_reg_ counterparts in pulmonary tissues. Importantly, changes in the microbiota of the lung have been found responsible for stimulating Th17-type responses that led to pulmonary fibrosis [6]. Vaccine studies have also elucidated the importance of Th17-type immunity. Vaccine-induced Th17-type responses were found to be necessary for protection against syncytial virus [1] and *H. Pylori* [7], largely via enhanced recruitment of neutrophils to infected tissues. Polarized findings of influenza vaccine research have shown that Th17-type responses are linked to both protection and exacerbation of lung pathology [8].

The balance of IL-17/IL-22 amidst a cytokine milieu and the microenvironment seems to determine whether Th17-type response plays a barrier-protective or proinflammatory role. Aptly, a new area of study is devoted to understanding the plasticity of Th17-type immune cells as their phenotypes can skew towards Th1-type, Th2-type, or immunosuppressive functions. Furthermore, Th17 CD4+ T cells and γδT cells can be maintained as long-lived effector memory cells in mucosal tissues. Thus, the induction of protective Th17-type responses in a given disease or vaccination setting depends on a more advanced knowledge of the distinct subsets of Th17-type cells and their functional responses.

In conclusion, there is significant evidence showing that Th17-type immunity and epithelial barrier functions have an important role in the immune response and inflammation of aging; however, the precise cellular and molecular mechanisms underlying altered Th17-type responses in aging humans remain to be elucidated. So far, targeted induction or dampening of Th17-type responses can be achieved using various methods (AhR/RORγt agonists or antagonists, PAMP/TLR adjuvants, microbial supplements) and their utilization is under active investigation. The health and diversity of our microbiome and how it influences Th17-type responses should also be of value for mucosal immunity in the context of aging. A clear understanding of epithelial barrier protective Th17-type responses will aid the development of targeted therapies, specifically tailored for the elderly.

## References

[1] Bystrom J, et al. Mediators Inflamm. 2015; 2015:205156. 10.1155/2015/205156 26101460 PMC4460252

[2] Walker EM, et al. Geroscience. 2019; 41:739–57. 10.1007/s11357-019-00099-7 31713098 PMC6925095

[3] Walker EM, et al. Front Immunol. 2021; 12:647398. 10.3389/fimmu.2021.647398 33717202 PMC7946846

[4] Kapil P, Merkel TJ. Curr Opin Immunol. 2019; 59:72–78. 10.1016/j.coi.2019.03.006 31078081 PMC6774807

[5] Orlov M, et al. J Immunol. 2020; 205:892–98. 10.4049/jimmunol.2000554 32651218 PMC7486691

[6] Yang D, et al. Immunity. 2019; 50:692–706.e7. 10.1016/j.immuni.2019.02.001 30824326

[7] Dixon BR, et al. Infect Immun. 2019; 87:e00363-19. 10.1128/IAI.00363-19 31427446 PMC6803329

[8] Frank K, Paust S. Front Cell Infect Microbiol. 2020; 10:425. 10.3389/fcimb.2020.00425 32974217 PMC7461885

